# The Finances of the Voluntary War Funds

**Published:** 1915-08-28

**Authors:** 


### The Hospital

The Workers' Newspaper of Administrative Medicine and Institutional Life, Administration, National Insurance and Health.

No. 1524, Vol. LVIII. SATURDAY, AUGUST 28, 1915. PRICE ONE PENNY

### THE FINANCES OF THE VOLUNTARY WAR FUNDS.

The financial aspect of the war is gradually compelling consideration both in its national and its personal relations. Its grave seriousness must be apparent to all. The complexities of the wider question can probably be appreciated only by experts, but a daily expenditure of three millions, with a daily income of three-quarters of a million, is, in essence, as simple as Mr. Micawber's well-known dilemma. Even those who have no head for figures are conscious of the increased cost of living, and the time is not distant when new taxes will add to the rigour of the lesson. Fine phrases about "silver bullets" and the like arouse in their day a glow of confidence in our national wealth and staying power, but the time appears when warlike metaphors have to be translated into terms of hard cash. It therefore becomes a very pertinent question whether our financial resources are being used to the best advantage, and whether they are being prepared for the strain which the future will certainly inflict on them. In its larger relations this problem is outside our sphere, but our constant concern with moneys raised voluntarily for philanthropic purposes may justify a suggestion bearing on the position of the various funds which the sufferings produced by the war have called into existence. Relatively to the national expenditure the total of these funds may no doubt be described as small. The amount, however, must be a considerable one, and, in any event, prudence and patriotism alike dictate that it shall be expended wisely, and that it shall be organised in accordance with sound financial principles.

The appeals to the public in the directions here contemplated have been numerous, and there can be no doubt that the response has been a generous one. When we learn that it is sufficient for a soldier to advertise himself as "lonely" to secure in the course of a few days several hundreds of letters and parcels, we can be certain that more stately appeals do not want for answers. There is the further proof, even more tangible, afforded by those of the larger funds which have announced in the Press the sums contributed to their support. Therefore it is reasonable to conclude that neither have organisations with a more limited ambition spoken to the public in vain. We now suggest that the time has come when these organisations should give a formal account of their stewardship—that they should, in other words, let it be known what moneys they have received, how these moneys have been spent, what balances remain in hand, and, so far as is possible, what expenditure is immediately contemplated. The majority of them have something like a twelvemonth's experience. It is prudent, it is in their own interest, that this experience in its financial terms should be publicly known.

The first reason we would urge in support of this suggestion is that, not unnaturally, subscribers like to know what has been done with their money. No one doubts the good faith of the administrators or is other than grateful to these for their self-sacrificing labours. All the same, it is a healthy instinct which prompts the inquiry—for what has the money been used? That it has been used generally for the purposes proposed is certain, but human nature likes something concrete and specific, particularly when the question is one of pounds, shillings, and pence. Especially is this true of individual specimens of human nature which, having given one subscription, are now asked for a second. Here we present the argument in the interests of the funds themselves. The balance-sheet need not be a rigorously severe one or set in too formal a fashion, but we have little doubt it would prove a most fruitful advertisement, and wise will be the organisations who call it to their aid.

A second support for our proposal may be found in the common experience that the practice of keeping a strict account of expenditure is an excellent aid to economy. The doctrine applies with as much force to charitable and patriotic undertakings as it does to the more severe exercises of commerce, and fortunate indeed is the fund which has on its direction some stern and unsentimental treasurer who insists that laudable motives shall not be divorced from prudent management. Probably there cannot be devised an apparatus more fruitful in wasteful expenditure than a committee of excellent persons set, with a full measure of emotion, on the prosecution of some lofty purpose. To speak, in such company, of accounts and vouchers and economies and balance sheets needs a courage not easily attained. Far be it from us to suggest that many of our war committees have failed to cultivate a more reasonable level. Yet we would fain see their virtues announced in the shape of figures, and those who provide this excellent example will have the happy consciousness that they are helping to encourage the others.

A third plea is associated with those blessed words "organisation" and "co-ordination." Rightly or wrongly, there is an impression abroad that certain of the war committees, though their appeals still reach us, have considerable funds in hand. Possibly they have evidence that these funds will be needed in the future, or even in the immediate future. Allowing this, it would still be well that the grounds for this anticipation should be known. The present is no time either for individual or corporate hoarding, except in so far as such action can be justified by a commonsense calculation of future demands. To bring all the war funds under the supervision of some central authority is, we recognise, an impossibility. As a nation we have elected for individualism, tempered by committees, and we must accept the disadvantages as well as the benefits of our choice. But our committees would be well advised to tell us what they are doing, and this as much in the interest of their continued success as well as out of respect for the general principles of sound finance.

